# Can We Cluster ICU Treatment Strategies for Traumatic Brain Injury by Hospital Treatment Preferences?

**DOI:** 10.1007/s12028-021-01386-y

**Published:** 2021-12-06

**Authors:** Iris E. Ceyisakar, Jilske A. Huijben, Andrew I. R. Maas, Hester F. Lingsma, Nikki van Leeuwen, CENTER-TBI participants and investigators, Cecilia Åkerlund, Krisztina Amrein, Nada Andelic, Lasse Andreassen, Audny Anke, Anna Antoni, Gérard Audibert, Philippe Azouvi, Maria Luisa Azzolini, Ronald Bartels, Pál Barzó, Romuald Beauvais, Ronny Beer, Bo-Michael Bellander, Antonio Belli, Habib Benali, Maurizio Berardino, Luigi Beretta, Morten Blaabjerg, Peter Bragge, Alexandra Brazinova, Vibeke Brinck, Joanne Brooker, Camilla Brorsson, Andras Buki, Monika Bullinger, Manuel Cabeleira, Alessio Caccioppola, Emiliana Calappi, Maria Rosa Calvi, Peter Cameron, Guillermo Carbayo Lozano, Marco Carbonara, Simona Cavallo, Giorgio Chevallard, Arturo Chieregato, Giuseppe Citerio, Hans Clusmann, Mark Coburn, Jonathan Coles, Jamie D. Cooper, Marta Correia, Amra Čović, Nicola Curry, Endre Czeiter, Marek Czosnyka, Claire Dahyot-Fizelier, Paul Dark, Helen Dawes, Véronique De Keyser, Vincent Degos, Francesco Della Corte, Hugo den Boogert, Bart Depreitere, Đula Đilvesi, Abhishek Dixit, Emma Donoghue, Jens Dreier, Guy-Loup Dulière, Ari Ercole, Patrick Esser, Erzsébet Ezer, Martin Fabricius, Valery L. Feigin, Kelly Foks, Shirin Frisvold, Alex Furmanov, Pablo Gagliardo, Damien Galanaud, Dashiell Gantner, Guoyi Gao, Pradeep George, Alexandre Ghuysen, Lelde Giga, Ben Glocker, Jagoš Golubovic, Pedro A. Gomez, Johannes Gratz, Benjamin Gravesteijn, Francesca Grossi, Russell L. Gruen, Deepak Gupta, Juanita A. Haagsma, Iain Haitsma, Raimund Helbok, Eirik Helseth, Lindsay Horton, Jilske Huijben, Peter J. Hutchinson, Bram Jacobs, Stefan Jankowski, Mike Jarrett, Ji-yao Jiang, Faye Johnson, Kelly Jones, Mladen Karan, Angelos G. Kolias, Erwin Kompanje, Daniel Kondziella, Evgenios Kornaropoulos, Lars-Owe Koskinen, Noémi Kovács, Ana Kowark, Alfonso Lagares, Linda Lanyon, Steven Laureys, Fiona Lecky, Didier Ledoux, Rolf Lefering, Valerie Legrand, Aurelie Lejeune, Leon Levi, Roger Lightfoot, Hester Lingsma, Andrew I. R. Maas, Ana M. Castaño-León, Marc Maegele, Marek Majdan, Alex Manara, Geoffrey Manley, Costanza Martino, Hugues Maréchal, Julia Mattern, Catherine McMahon, Béla Melegh, David Menon, Tomas Menovsky, Ana Mikolic, Benoit Misset, Visakh Muraleedharan, Lynnette Murray, Ancuta Negru, David Nelson, Virginia Newcombe, Daan Nieboer, József Nyirádi, Otesile Olubukola, Matej Oresic, Fabrizio Ortolano, Aarno Palotie, Paul M. Parizel, Jean-François Payen, Natascha Perera, Vincent Perlbarg, Paolo Persona, Wilco Peul, Anna Piippo-Karjalainen, Matti Pirinen, Dana Pisica, Horia Ples, Suzanne Polinder, Inigo Pomposo, Jussi P. Posti, Louis Puybasset, Andreea Radoi, Arminas Ragauskas, Rahul Raj, Malinka Rambadagalla, Isabel Retel Helmrich, Jonathan Rhodes, Sylvia Richardson, Sophie Richter, Samuli Ripatti, Saulius Rocka, Cecilie Roe, Olav Roise, Jonathan Rosand, Jeffrey V. Rosenfeld, Christina Rosenlund, Guy Rosenthal, Rolf Rossaint, Sandra Rossi, Daniel Rueckert, Martin Rusnák, Juan Sahuquillo, Oliver Sakowitz, Renan Sanchez-Porras, Janos Sandor, Nadine Schäfer, Silke Schmidt, Herbert Schoechl, Guus Schoonman, Rico Frederik Schou, Elisabeth Schwendenwein, Charlie Sewalt, Ranjit D. Singh, Toril Skandsen, Peter Smielewski, Abayomi Sorinola, Emmanuel Stamatakis, Simon Stanworth, Robert Stevens, William Stewart, Ewout W. Steyerberg, Nino Stocchetti, Nina Sundström, Riikka Takala, Viktória Tamás, Tomas Tamosuitis, Mark Steven Taylor, Braden Te Ao, Olli Tenovuo, Alice Theadom, Matt Thomas, Dick Tibboel, Marjolein Timmers, Christos Tolias, Tony Trapani, Cristina Maria Tudora, Andreas Unterberg, Peter Vajkoczy, Shirley Vallance, Egils Valeinis, Zoltán Vámos, Mathieu van der Jagt, Gregory Van der Steen, Joukje van der Naalt, Jeroen T. J. M. van Dijck, Inge A. van Erp, Thomas A. van Essen, Wim Van Hecke, Caroline van Heugten, Dominique Van Praag, Ernest van Veen, Thijs Vande Vyvere, Roel P. J. van Wijk, Alessia Vargiolu, Emmanuel Vega, Kimberley Velt, Jan Verheyden, Paul M. Vespa, Anne Vik, Rimantas Vilcinis, Victor Volovici, Nicole von Steinbüchel, Daphne Voormolen, Petar Vulekovic, Kevin K. W. Wang, Daniel Whitehouse, Eveline Wiegers, Guy Williams, Lindsay Wilson, Stefan Winzeck, Stefan Wolf, Zhihui Yang, Peter Ylén, Alexander Younsi, Frederick A. Zeiler, Veronika Zelinkova, Agate Ziverte, Tommaso Zoerle

**Affiliations:** 1 Center for Medical Decision Making, Department of Public Health, Erasmus Medical Center, PO Box 2040, 3000 CA Rotterdam, The Netherlands; 2 Department of Neurosurgery, Antwerp University Hospital, University of Antwerp, Drie Eikenstraat 655, 2650 Antwerp, Belgium

**Keywords:** Provider profiling, Between-hospital variation, Comparative effectiveness research, Traumatic brain injury

## Abstract

**Background:**

In traumatic brain injury (TBI), large between-center differences in treatment and outcome for patients managed in the intensive care unit (ICU) have been shown. The aim of this study is to explore if European neurotrauma centers can be clustered, based on their treatment preference in different domains of TBI care in the ICU.

**Methods:**

Provider profiles of centers participating in the Collaborative European Neurotrauma Effectiveness Research in TBI study were used to assess correlations within and between the predefined domains: intracranial pressure monitoring, coagulation and transfusion, surgery, prophylactic antibiotics, and more general ICU treatment policies. Hierarchical clustering using Ward’s minimum variance method was applied to group data with the highest similarity. Heat maps were used to visualize whether hospitals could be grouped to uncover types of hospitals adhering to certain treatment strategies.

**Results:**

Provider profiles were available from 66 centers in 20 different countries in Europe and Israel. Correlations within most of the predefined domains varied from low to high correlations (mean correlation coefficients 0.2–0.7). Correlations between domains were lower, with mean correlation coefficients of 0.2. Cluster analysis showed that policies could be grouped, but hospitals could not be grouped based on their preference.

**Conclusions:**

Although correlations between treatment policies within domains were found, the failure to cluster hospitals indicates that a specific treatment choice within a domain is not a proxy for other treatment choices within or outside the domain. These results imply that studying the effects of specific TBI interventions on outcome can be based on between-center variation without being substantially confounded by other treatments.

**Trial registration:**

We do not report the results of a health care intervention.

**Supplementary Information:**

The online version contains supplementary material available at 10.1007/s12028-021-01386-y.

## Introduction

Traumatic brain injury (TBI) remains a major global health issue, being one of the leading causes of mortality and disability with 2.5 million reported cases each year within the European Union and United Kingdom [1–3].

The primary injury is irreversible, and the main focus of treatment is on avoiding and limiting secondary brain damage. In patients with severe TBI, this is often informed by intracranial pressure (ICP) or brain-metabolic monitoring. Previous studies have debated monitoring and treatment choices in TBI [4], and evidence underpinning monitoring and treatment recommendations is relatively weak.

This uncertainty is reflected in large between-center differences in processes and outcomes for patients treated in the intensive care unit (ICU) after TBI [5, 6]. The differences in treatment policy can be exploited to study treatment effectiveness in comparative effectiveness research (CER). One approach to CER is to identify the most effective treatment, by comparing hospitals’ treatment choices and relating these to their outcomes. In recent years, this approach has gained popularity in TBI as a complementary approach to the evidence base provided by randomized controlled trials [7]. CER can be used to identify a causal relationship between a treatment and outcome if known and unknown confounders can be adequately adjusted for and if the treatment under investigation is not correlated with other treatment policies. To date, it is unknown whether certain treatment strategies in patients with TBI are related. Such knowledge would be essential when comparing outcomes on a hospital level within the framework of CER to study whether differences in outcomes can be attributed to the separate interventions.

If, on the other hand, multiple treatment choices are correlated, it gives the possibility to group these together and identify hospitals with, for example, a more aggressive treatment strategy. Conclusions could then only be drawn on a very general level: whether a more aggressive or a more passive treatment strategy is more effective. Within the framework of CER, however, this would make it impossible to study specific treatments and their effect on outcome because some specific treatment aspects within the strategy may be beneficial and others even harmful.

Focusing on the domains of ICP monitoring, prophylactic antibiotics, transfusion targets, and general ICU management, our aim was to investigate correlations between treatment policies and to explore if European neurotrauma centers can be clustered based on their treatment strategy in patients with TBI.

## Methods

### Collaborative European Neurotrauma Effectiveness Research in TBI Study

The Collaborative European NeuroTrauma Effectiveness Research in TBI (CENTER-TBI) study is a prospective longitudinal multicenter observational study conducted across Europe and Israel (ClinicalTrials.gov ref. NCT02210221) [8]. CENTER-TBI aims to better characterize and describe TBI in a European context and to further advance the care of patients with TBI within the broader international framework of the International Initiative for TBI Research (https://intbir.nih.gov/).

Principal investigators of each participating center in this study received questionnaires about the structures (type of facilities and equipment, the qualifications of medical staff and their organizations) and processes (treatment policies in different phases of TBI care) of their center: the provider profiling questionnaires [9]. Participants were explicitly asked for their general policy rather than for individual treatment preferences. General policy was defined as ‘‘the way the large majority of patients (> 75%) with a certain indication would be treated.’’ Detailed information about the content, development, and validation of the original 321 questions can be found in an earlier publication [9]. Baseline characteristics for centers were described using frequencies and percentages.

### Predefined Treatment Domains

We selected 58 questions on the basis of expert consensus concerning care in the ICU setting, covering the domains of coagulation and transfusion, neurosurgery, ICP monitoring, prophylactic antibiotics, and general management. The selected questions were chosen before the analysis on the basis of clinical relevance. Follow-up, conditional questions (“if you answered A, then specify…”) and all open questions were excluded, aiming to obtain a standardized overview of treatment approaches.

Questions from predefined domains were chosen to enable stratification of hospitals over multiple domains. To determine possible underlying treatment strategies, polychoric correlation coefficients between questions were calculated [10]. Correlations were visualized with correlation plots, using only absolute values (between 0 and 1), as any negative correlations were as relevant as positive correlations in determining treatment strategies. Missing answers were disregarded for calculation of the correlation. Of the 58 questions, 44 questions were complete, and the other 12 had up to 8 out of 66 answers missing. We looked at correlations of questions *within* the predefined domains (exploring consistency in treatment policies within a specific domain) as well as correlations *between* the domains (exploring interdependencies of treatments between domains).

### Regrouping of Questions

After correlations were determined, the questions were grouped based on the data, ignoring the previously defined domains, with an hierarchical cluster analysis using Ward’s minimum variance method, to group together the questions with the highest similarity [11]. This is an agglomerative clustering method in which the data points (questions) are clustered (across all domains) in different steps until only questions with the greatest similarity form a cluster. We used the Bayesian inference criterion for *k*-means to determine the number of clusters that were to be formed [12, 13].

### Heat Maps

By using the same clustering algorithm, heat maps were made for each newly formed group of questions. Heat maps allow for the recognition of patterns in the preference of hospitals and made it possible to determine whether we could discern certain types of hospitals. This was performed on all complete cases: centers with missing answers within the cluster of questions were disregarded. For cluster two, 53 of the 66 participating hospitals were included in the heat map; for cluster five, 47 of the hospitals were included, and all other clusters included all participating hospitals. All analysis were performed in R version 3.3.0 using the following packages: pheatmap, RColorBrewer, foreign, cluster, corrplot, dplyr, and fmsb [14–21].

## Results

### CENTER-TBI Study

Provider profiling questionnaires were completed in 66 centers (97% response rate), mainly by intensivists (*n* = 33, 50%) and neurosurgeons (*n* = 23, 35%), but otherwise by administrative staff (*n* = 11, 17%), neurologists (*n* = 5, 8%), anesthesiologists (*n* = 5, 8%), and a trauma surgeon (*n* = 1, 2%). The majority of these centers had an academic affiliation (*n* = 60, 91%). The center characteristics are described in Supplementary Table 1 and in more detail in a previous publication [9].

### Correlation within and Between Domains

**Fig. 1:**
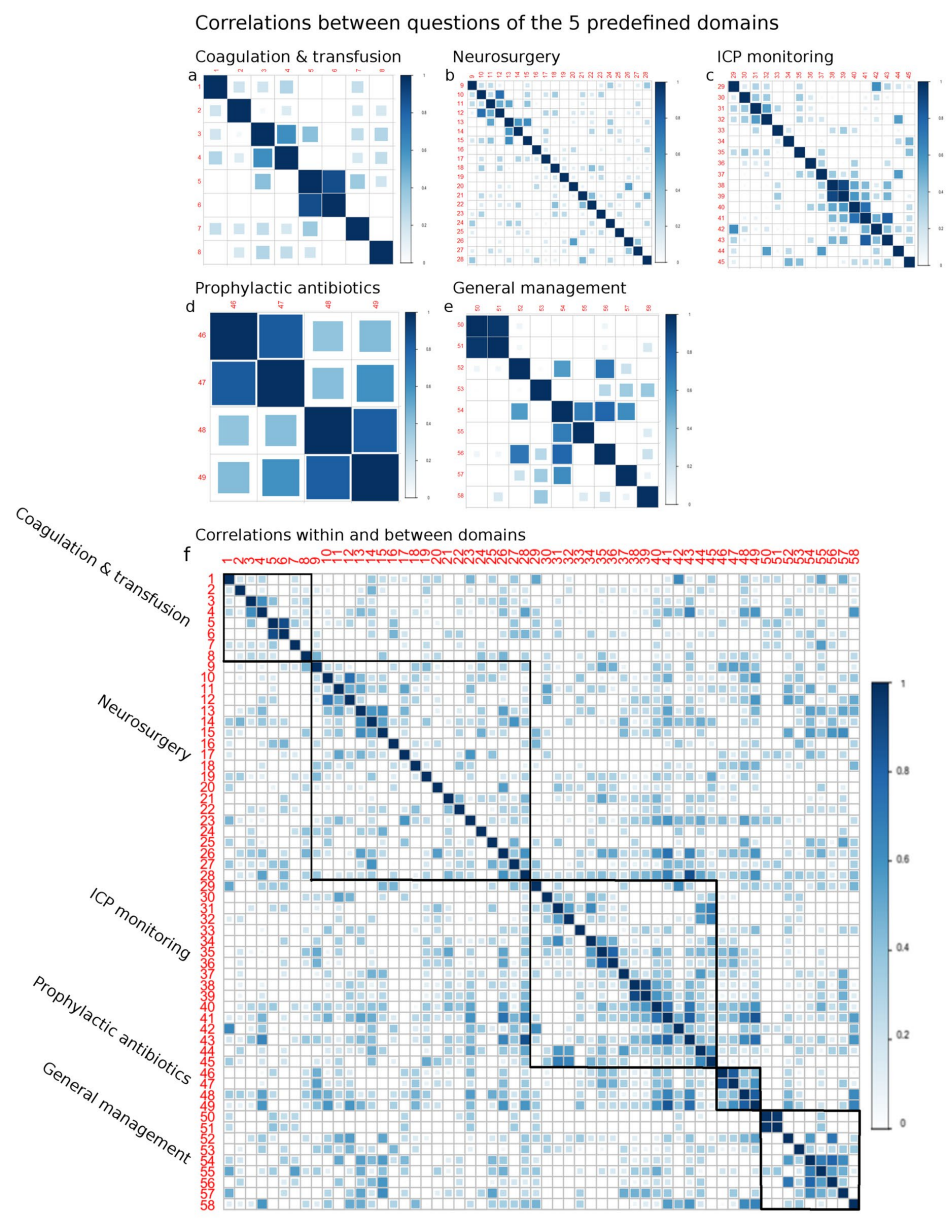
Correlation plot showing correlations between questions, grouped (squares) to show the five predefined domains: intracranial pressure (ICP) monitoring (**a**), coagulation and transfusion (**b**), surgery (**c**), prophylactic antibiotics (**d**), and more general ICU treatment policies (**e**), and correlations within and between the predefined domains (**f**). The correlations were calculated with Pearson correlations, and a higher correlation is visualized as a darker blue. *ICU* intensive care unit

Correlation between treatment policies within the predefined domains was variable (Fig. 1a–e). Correlations within the domain of prophylactic antibiotics (mean correlation coefficient = 0.6, range 0.4–0.8) ranged from moderate to strong, but questions were based on only one very specific topic. The correlation within the other domains was shown to be much lower (Table 1). Correlations between domains were lower, with mean correlation coefficients of 0.2 for each domain correlated with all other domains (Table 1 and Fig. 1f).

**Table 1:** Overview of the correlation coefficient calculated for questions within and between predefined domains

Domain	Mean (SD)	Min	Max	Number of questions
Correlation coefficients for correlation between questions *within* the predefined domains				
Coagulation & transfusion targets	0.2 (0.2)	0	0.9	8
Neurosurgery	0.2 (0.2)	0	0.8	19
ICP monitoring	0.3 (0.2)	0	0.9	17
Prophylactic antibiotics	0.6 (0.2)	0.4	0.8	4
General management	0.3 (0.2)	0	1	9
Correlation coefficients for questions *between* the predefined domains				
Coagulation & transfusion targets versus rest	0.2 (0.1)	0	0.7	
Neurosurgery versus rest	0.2 (0.2)	0	0.9	
ICP monitoring versus rest	0.2 (0.2)	0	0.9	
Prophylactic antibiotics versus rest	0.2 (0.2)	0	0.9	
General management versus rest	0.2 (0.1)	0	0.7	

We used the Pearson correlation coefficient to determine correlations within domains (upper panel) and between domains (lower panel)

*ICP* intracranial pressure, *Max* maximum, *Min* minimum, *SD* standard deviation

### Data-Driven Cluster Analysis

**Fig. 2:**
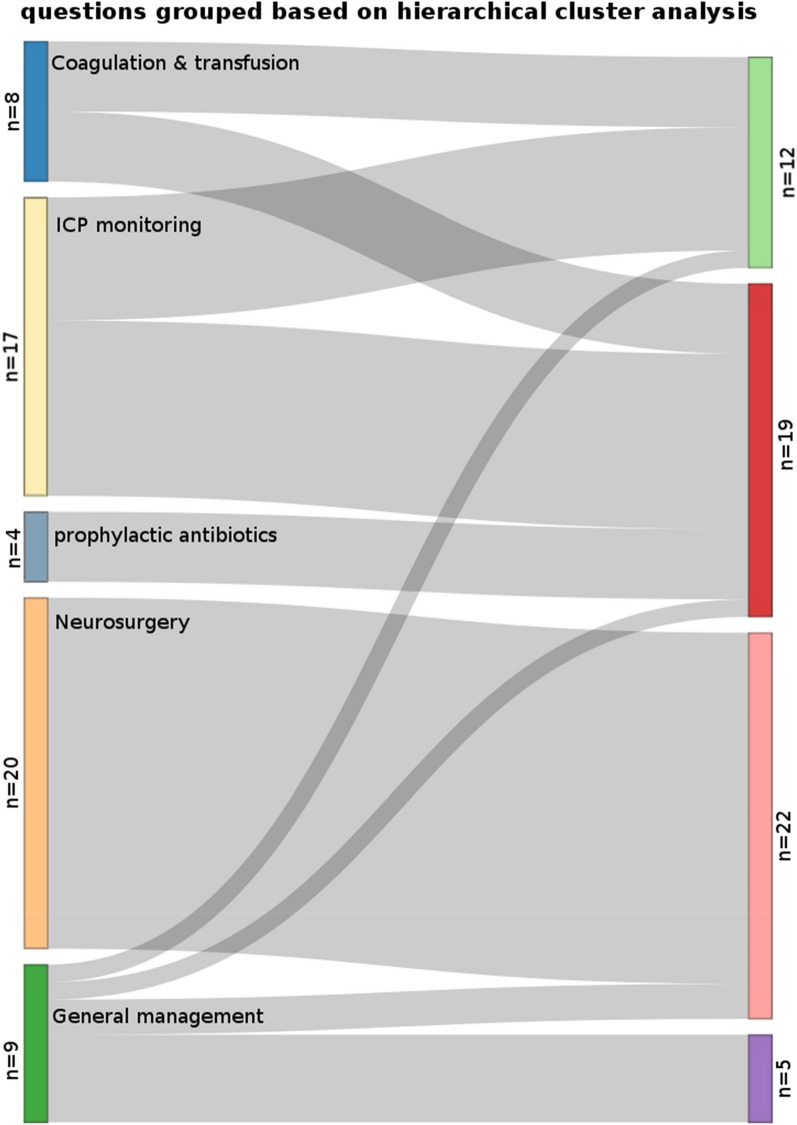
Sankey diagram showing regrouping of questions according to hierarchical clustering. On the left, questions are grouped according to what was decided to be clinically relevant. On the right, the questions are regrouped, and the shifting of questions is visualized, with a thicker gray line indicating a larger number of questions. ICP, intracranial pressure

The cluster analysis revealed four clusters, one fewer compared with the clinically determined domains (Fig. 2). The grouping remained very similar to the predefined domains, especially for the original domain of neurosurgery. A few questions did correlate with other subdomains, mainly due to overlap in topic of the questions. For example, the question, “Is a coagulation panel assessed prior to insertion of an ICP monitoring device?” could span the domains of coagulation and transfusion as well as ICP monitoring (Supplementary Table 2).

### Grouping of Hospitals

**Fig. 3:**
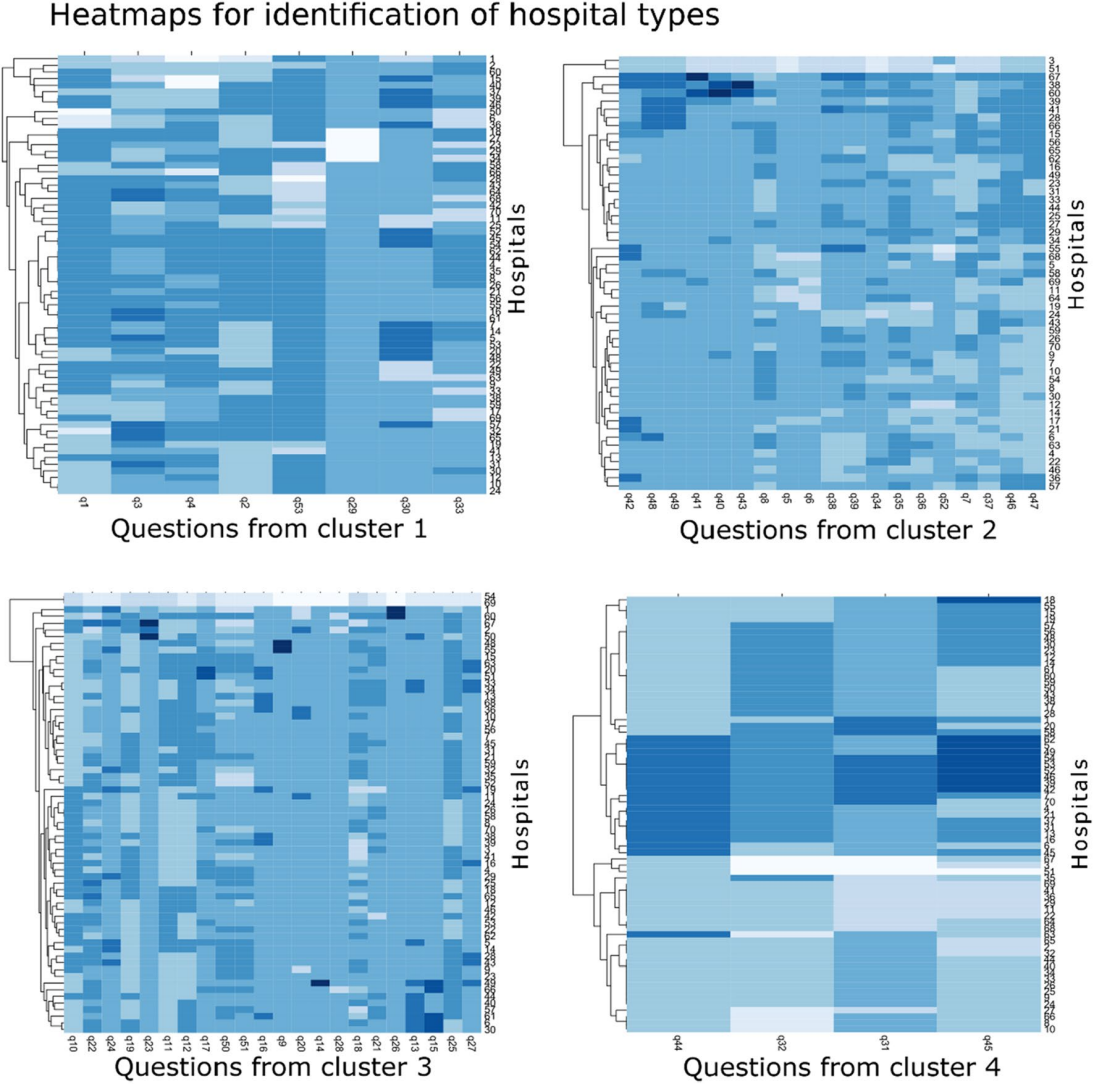
Heat maps showing similarities between hospitals in answers given to questions within the previously determined clusters of questions. This was based on the hierarchical cluster analyses. The colors in the heat map relate to the specific answers: within a column, the same color indicates that two hospitals have given the same answer to the corresponding question. *ICP* intracranial pressure

No hospital types were discernable in the heat maps made for each cluster of questions (Fig. 3, for the heat map of all the questions see Suppl. Figure 2). The heat maps served as a visual indication of the possibility to cluster the hospitals. Based on the visualization of these data, we have decided that further clustering should not be done. Although most similar questions had been grouped together, heat maps showed no clear pattern in the preferences of the hospitals, indicating that they could not be grouped based on their treatment tendencies.

## Discussion

This study aimed to group European neurotrauma centers into clusters based on their treatment preference in patients with TBI in a variety of domains. Hospitals could not be clustered based on their reported choices of treatment within the five domains of the provider profiling questionnaires. These results imply that it is unlikely that hospitals can be categorized as a certain type of hospital based on the treatment strategies they follow across multiple domains of monitoring and treatment in patients with TBI.

The lack of evident treatment policies across multiple domains might be explained by a lack of strong evidence of the effectiveness of certain treatments, leading to weak guideline recommendations, which may cause heterogeneity in treatment strategies across Europe [22]. However, it could also be the result of more individualized medicine, in which case treatment strategies are based on the individual patient and monitoring characteristics [1]. TBI is a complex heterogeneous syndrome that might not be captured with a single treatment strategy. With the advanced monitoring devices and the range of brain and system targeted therapies available, variation between centers in treatment strategies is likely.

For future statistical analyses, our finding that the questions correlated mainly within their previously defined subdomain implies that all elements of TBI treatment can, and have to, be analyzed separately rather than combining different domains when relations between treatment and outcome are explored. The correlation of some questions with questions from a different domain could be attributed to overlap in the subject of the questions. Other reasons for correlations with other domains could be dependent on who is responsible for the decisions being made: for example, decisions for treatment of the patient are made by the neurosurgeon would have a higher chance of correlating with other decisions made by that neurosurgeon.

Based on our study, we may conclude that future CER analyses will be likely to measure a direct effect of one intervention on outcome instead of a general effect of multiple treatment effects. This is important knowledge to continue CER research within TBI, in which outcomes between centers are compared to find underlying differences in treatment. Although unmeasured confounders will always have to be considered, knowing that multiple treatments are not interdependent is a first step in further elucidating the effects of treatment choices. This study has its strengths and limitations. This study was conducted in multiple neurotrauma centers across Europe. The development and dissemination of the questionnaires was done in different phases. Two methods were used to determine relations between and within certain treatment strategy domains. With hierarchical cluster analyses, we confirmed the results of correlation analyses. However, our study also has its limitations; in a survey study using provider profiling questionnaires, centers only indicate their treatment strategy and do not provide an objective measure of real-time practice. This could overestimate or underestimate the use of general policies. The centers included in this study are mostly academic medical centers, and a more heterogeneous group of care providers could have potentially shown a clearer division in hospital types. Previous studies from CENTER-TBI show that, even within the sample of mostly academic centers, substantial practice variation exists [23–25].

Further, the study is focused on hospitals in Europe, and it is possible that these findings cannot be extrapolated to other large regions, such as the United States.

Possibly better suited for the purpose of grouping hospitals would be a questionnaire that is more specific. Future research using a more detailed questionnaire might be a solution to increase reliability of indicated treatment preferences. More targeted questions could allow for a better and more thorough understanding. This would give insight into why decisions are made and by whom. However, the better we understand and the more specific the information is, the harder it will be to visualize, generalize, and simplify enough to be able to present it graphically.

This is the first study that studied underlying relations in treatment strategies, and these results need to be confirmed in other studies.

## Conclusions

We found correlations in treatment policies within domains, especially for neurosurgical interventions, but no evidence that hospitals could be clustered, indicating that a specific treatment choice within a domain is not a proxy for other treatment choices within or outside the domain. Because we did not find an indication that some centers, in general, were more eager to treat or reach higher treatment intensity levels overall, future TBI analyses should be conducted per specific treatment item instead of per treatment domain. Furthermore, within the CER paradigm, this implies that analyzing effects of an intervention on outcome is likely to measure a direct effect of that intervention without being substantially confounded by a general effect of multiple treatments.

## Supplementary Information

Below is the link to the electronic supplementary material.

Supplementary Figure 1. Correlation plot-showing correlations between questions, after questions have been grouped according to hierarchical clustering (TIFF 3344 KB)

Supplementary Figure 2. Heatmaps showing similarity between hospitals in answers given to question based on the hierarchical cluster analyses. The colors in the heatmap relate to the specific answers: within a column the same color indicates that two hospitals have given the same answer to the corresponding question (TIFF 40917 KB)

Supplementary file3 (DOCX 23 KB)

## Data Availability

The datasets generated and/or analysed during the current study are not publicly available because participants gave no consent for data sharing. The datasets generated and/or analysed during the current study are not publicly available because participants gave no consent for data sharing.
