# Supplementary figures and images for: Can We Cluster ICU Treatment Strategies for Traumatic Brain Injury by Hospital Treatment Preferences?
Source: Neurocrit Care. 2021 Dec 6;36(3):846–56. doi: 10.1007/s12028-021-01386-y (PMC9110448; doi:10.1007/s12028-021-01386-y)

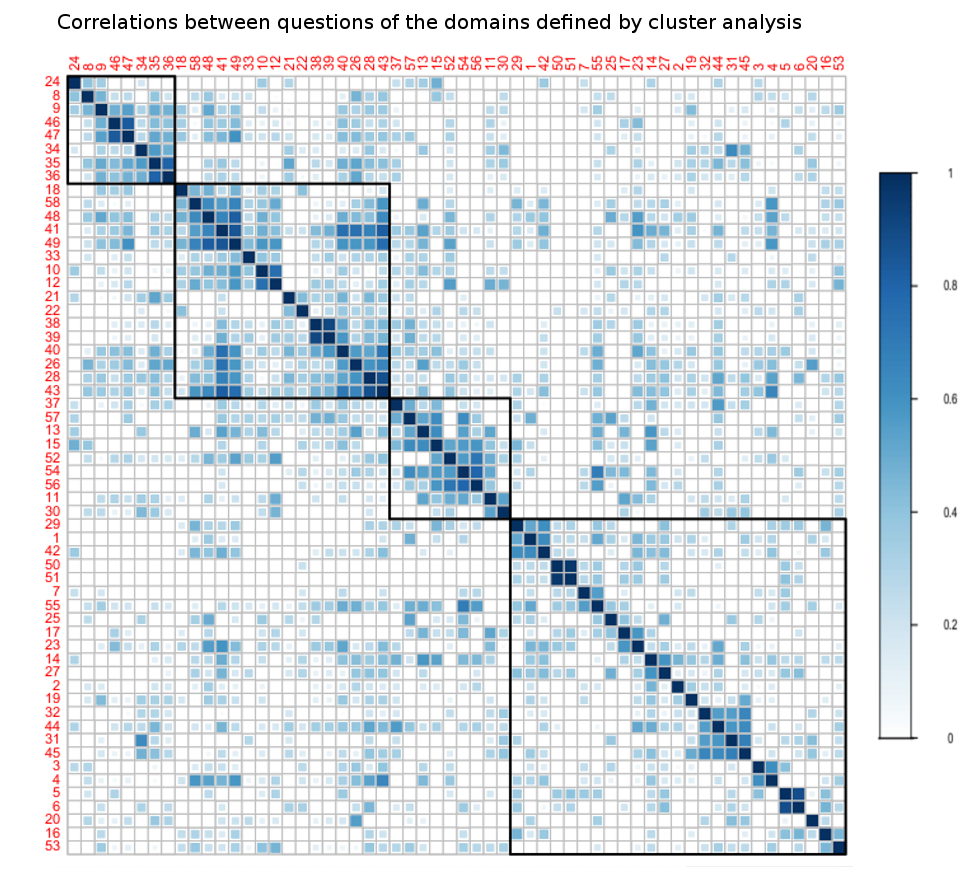

Supplement: Supplementary file 1 — Supplementary Figure 1. Correlation plot-showing correlations between questions, after questions have been grouped according to hierarchical clustering (TIFF 3344 KB) [file 12028_2021_1386_MOESM1_ESM.tiff]

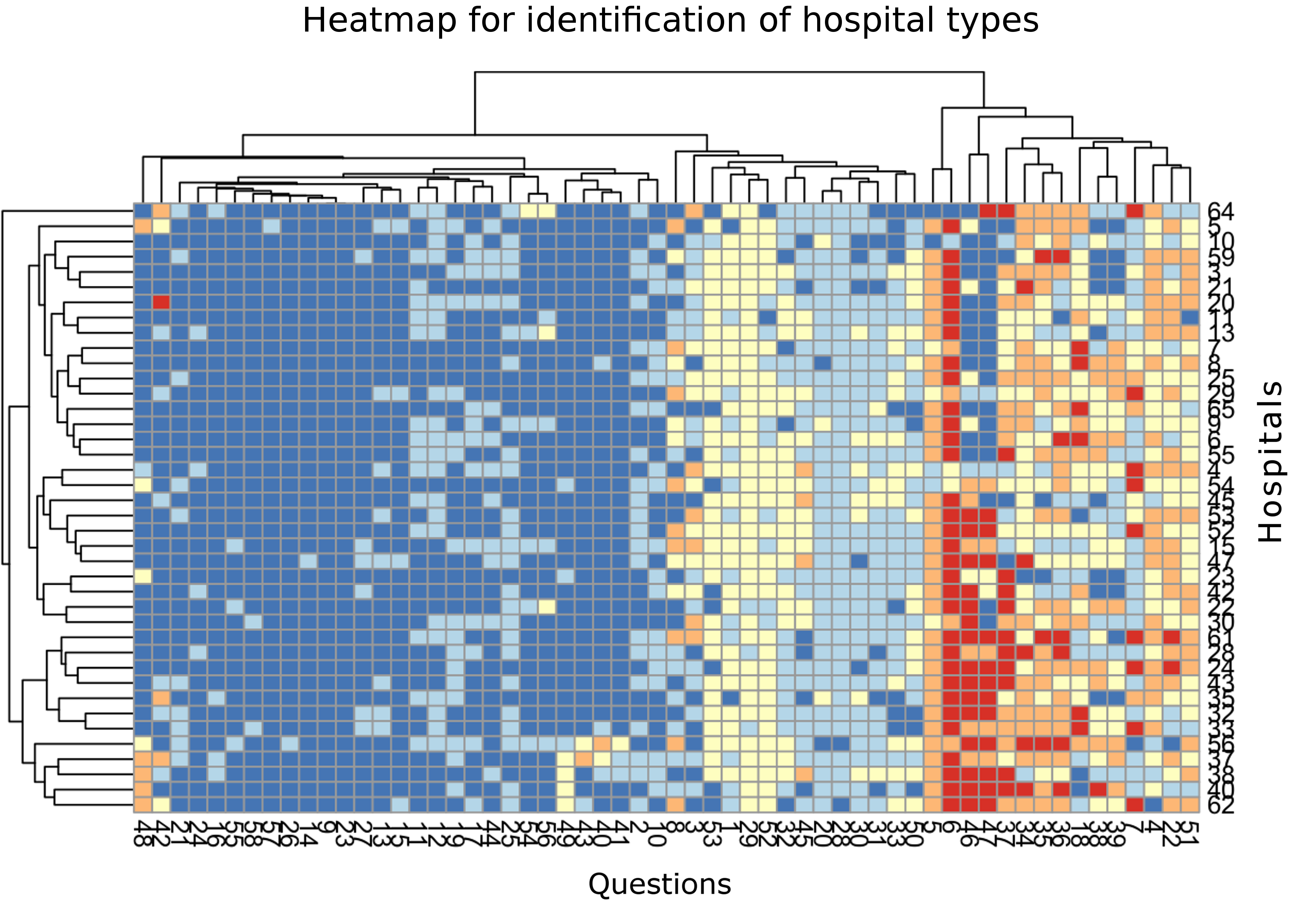

Supplement: Supplementary file 2 — Supplementary Figure 2. Heatmaps showing similarity between hospitals in answers given to question based on the hierarchical cluster analyses. The colors in the heatmap relate to the specific answers: within a column the same color indicates that two hospitals have given the same answer to the corresponding question (TIFF 40917 KB) [file 12028_2021_1386_MOESM2_ESM.tiff]
