# Supplementary material for: Can We Cluster ICU Treatment Strategies for Traumatic Brain Injury by Hospital Treatment Preferences?
Source: Neurocrit Care. 2021 Dec 6;36(3):846–56. doi: 10.1007/s12028-021-01386-y (PMC9110448; doi:10.1007/s12028-021-01386-y)
Supplement: Supplementary file 3 — Supplementary file3 (DOCX 23 KB) [file 12028_2021_1386_MOESM3_ESM.docx]

## Supplementary Tables

| **Baseline characteristics centers** | **N (%)*** |
| --- | --- |
| Academic hospital (vs. non-Academic) | n = 60, 91% |
| questionnaire completed by |  |
| - Intensivists | n = 33, 50% |
| - Neurosurgeons | n = 23, 35% |
| - administrative staff | n = 11, 17% |
| - neurologists | n = 5, 8% |
| - anesthesiologists | n = 5, 8% |
| - trauma surgeon | n = 1, 2% |
| Trauma center designation |  |
| - Level I | n = 45, 69% |
| - Level II | n = 4, 6% |
| - Level III | n = 1, 2% |
| - No designation / NA | n = 15, 23% |
| The availability of a dedicated neuro ICU | n = 39, 59% |
|  |  |
| Suppl. table 1. |  |
|  |  |
|  |  |

| Q | **Questions used for analysis & the domain they belong to** |
| --- | --- |
|  | Coagulation & transfusion |
| 1 | **How often is DVT prophylaxis used?**   - Never (0-10%) - Rarely (10-30%) - Sometimes (30-70%) - Frequently (70-90%), - Always (90-100%) |
| 2 | **Does the Intensive Care Unit (ICU) protocol specify a target goal for hemoglobin concentration?**   - Yes - No |
| 3 | **Do you have a transfusion target in patients with Traumatic Brain Injury (TBI) in the acute phase?**   - >100 g/l or 6 mmol/l - Between 90g/l or 5.5 mmol/l and 100 g/l or 6 mmol/l - between 80 g/l or 5 mmol/l and 90 g/l or 5.5 mmol/l - Between 70 g/l or 4.0 mmol/l and 80 g/l or 5 mmol/l |
| 4 | **What is your transfusion target in patients with non-neurological critical illness?**   - >100 g/l or 6 mmol/l - Between 90g/l or 5.5 mmol/l and 100 g/l or 6 mmol/l - between 80 g/l or 5 mmol/l and 90 g/l or 5.5 mmol/l - Between 70 g/l or 4.0 mmol/l and 80 g/l or 5 mmol/l |
|  |  |
|  | **Is a coagulation panel assessed prior to insertion of an ICP monitoring device?** |
| 5 | Ventricular catheter   - Never (0-10%) - Rarely (10-30%) - Sometimes (30-70%) - Frequently (70-90%), - Always (90-100%) |
| 6 | Parenchymal sensor   - Never (0-10%) - Rarely (10-30%) - Sometimes (30-70%) - Frequently (70-90%), - Always (90-100%) |
|  |  |
| 7 | **What is considered a minimum platelet count for insertion of a ventricular catheter in your Intensive Care Unit?**   - >150K - >100K - > 80 K - >50K - Variable, depends on surgeon - No minimum |
| 8 | **What is considered the minimum INR for safe placement of a ventricular catheter in your Intensive Care Unit (ICU)?**   - <1.4 - <1.3 - <1.2 - Variable, depending on surgeon - No minimum - Other |
|  | Neurosurgery |
|  | **In your hospital, is there 24/7 qualified neurosurgical coverage?**  *Select all that apply* |
| 9 | No |
| 10 | There is 24/7 in-house availability of a qualified neurosurgeon |
| 11 | There is 24/7 in-house availability of a neurosurgical trainee in residency training |
| 12 | Qualified neurosurgeons are on call and will arrive within 30 minutes |
| 13 | Neurosurgical trainees in residency training are on call and will arrive within 30 minutes |
| 14 | Qualified neurosurgeons are on call and will arrive in more than 30 minutes |
| 15 | There is 24/7 access to a qualified neurosurgeon by telecommunication / phone. Qualified neurosurgeons are however not 24/7 in-house or on call |
|  |  |
| 16 | **What is the general policy with regard to management of extremity (limb) fractures in patients with severe Traumatic Brain Injury (TBI) ?**   - Damage control: We focus on the TBI. All extremity fractures are stabilized, but definitive treatment delayed - Definitive care: We try to operate/fixate the extremity fractures as soon as possible |
|  |  |
| 17 |  |
| 18 | **Is the decision on surgery in acute SDH influenced by age?**   - Never (0-10%) - Rarely (10-30%) - Sometimes (30-70%) - Frequently (70-90%), - Always (90-100%) |
| 19 | **Are there acute SDH volume/ thickness thresholds above which your protocol/institutional practice advises surgery (i.e. evacuation)?**   - No - Yes |
| 20 | **Can decompressive craniectomy be added to the surgical evacuation of the acute SDH?**  o Yes, standard and routinely in every patient  o Yes, but dependent on intraoperative findings  o No, sometimes delayed in a second procedure in case of uncontrollable ICP o No, never |
| 21 | **The general policy in your institute for management of intraparenchymal mass lesions (contusions)**   - - Pre-emptive surgery to prevent deterioration   - Delayed surgery only after deterioration including intracranial hypertension   - Variable, depending on surgeon   - Other |
| 22 | **In case of refractory intracranial hypertension / pressure, how often do you use decompressive craniectomy in patients with severe Traumatic Brain Injury (TBI)?**   - Never (0-10%) - Rarely (10-30%) - Sometimes (30-70%) - Frequently (70-90%), - Always (90-100%) |
| 23 | **Are there ICP thresholds (eg > 20, 25, 30 mm Hg), above which you would consider delayed decompressive craniectomy?**   - 1. No, I would never perform a decompressive craniectomy   o No, I do not use ICP values in the decision to perform a decompressive craniectomy  o Yes |
|  |  |
|  | **What are indications for decompressive craniectomy?**  *Select all that apply* |
| 24 | ICP not monitored, but CT evidence of raised ICP |
| 25 | Not directly planned, but decided on because of intra-operative brain swelling |
| 26 | Routinely performed with every acute SDH or contusion evacuation |
| 27 | Pre-emptive approach to treatment of (suspected) raised ICP (not last resort) |
| 28 | Raised ICP, refractory to medical management (last resort) |
|  | ICP monitoring |
|  | **What are indications for ICP monitoring in your hospital?**  o Considered not important in decision making  o Only in the presence of other risk factors  o General Policy  provide us the general clinical practice at your centre for following situations |
| 29 | GCS ≤ 8 and CT abnormalities |
| 30 | GCS ≤ 8 without CT abnormalities |
| 31 | GCS 9-12 with contusion |
| 32 | Inability to assess a patient with CT abnormalities clinically (e.g. sedation, surgery etc.) |
| 33 | Intraventricular hemorrhage |
|  |  |
|  | **What are reasons for NOT monitoring ICP at your Intensive Care Unit?**  o Considered not important in decision making  o Only in the presence of other risk factors  o General Policy  provide us the general clinical practice at your centre for following situations |
| 34 | Glasgow Coma Scale (GCS) > 8 |
| 35 | No radiological signs of raised ICP |
| 36 | Risk of raised ICP considered low |
| 37 | Patient considered unsalvageable |
| 38 | Coagulopathy (non-drug related) |
| 39 | Use of anticoagulants or platelet aggregation inhibitors |
| 40 | No device available |
| 41 | Not local policy to monitor ICP |
| 42 | We adhere to a protocol in which treatment is based on imaging and clinical examination |
| 43 | Too costly |
|  |  |
|  | **When a patient with polytrauma and minor intracranial pathology (which would not otherwise indicate ICP monitoring) requires extracranial surgery which is not life-saving, in the acute phase after trauma, do you:** |
| 44 | place an ICP monitor and allow surgery to proceed   - No - Yes |
|  |  |
| 45 | **In polytrauma patients with a Glasgow Coma Scale (GCS) > 8 and small but not severe initial CT abnormalities, who require mechanical ventilation for a number of days because of extracranial injuries, we apply ICP monitoring:**   - Never (0-10%) - Rarely (10-30%) - Sometimes (30-70%) - Frequently (70-90%), - Always (90-100%) |
|  | prophylactic antibiotics |
|  | **Are prophylactic antibiotics given prior to ICP monitor insertion?** |
| 46 | Ventricular catheter   - Never (0-10%) - Rarely (10-30%) - Sometimes (30-70%) - Frequently (70-90%), - Always (90-100%) |
| 47 | Parenchymal sensor   - Never (0-10%) - Rarely (10-30%) - Sometimes (30-70%) - Frequently (70-90%), - Always (90-100%) |
|  |  |
|  | **Are prophylactic antibiotics continued after ICP monitoring insertion?** |
| 48 | Ventricular catheter   - Never (0-10%) - Rarely (10-30%) - Sometimes (30-70%) - Frequently (70-90%), - Always (90-100%) |
| 49 | Parenchymal sensor   - Never (0-10%) - Rarely (10-30%) - Sometimes (30-70%) - Frequently (70-90%), - Always (90-100%) |
|  | General management |
|  | **Are the Brain Trauma Foundation guidelines followed for:** |
| 50 | Epidural hematoma (EDH) management? |
| 51 | Acute Subdural hematoma (SDH) management? |
| 52 | Management of intraparenchymal mass lesions (contusions)? |
|  |  |
|  | **Who inserts the catheter/probes for ICP monitoring?** |
| 53 | Neurosurgeon   - Never - Rarely/ Exceptional - General Policy |
| 54 | Neurosurgical resident   - Never - Rarely/ Exceptional - General Policy |
| 55 | Intensivist   - Never - Rarely/ Exceptional - General Policy |
| 56 | Intensivist resident   - Never - Rarely/ Exceptional - General Policy |
| 57 | Neurointensivist   - Never - Rarely/ Exceptional - General Policy |
| 58 | Non-neurosurgical surgeon, Physician assistant / nurse practitioner   - Never - Rarely/ Exceptional - General Policy |

Suppl. Table 2.
